# Obituary

**Published:** 1869-07-01

**Authors:** 


### OBITUARY.

DIED, in Philadelphia, on the 1st of April, after a protracted illness, ROBLEY DUNGLISON, M.D., Emeritus Professor of the Institutes of Medicine in Jefferson Medical College, and late Dean of that Faculty.

Few physicians in this country have established a wider reputation than Dr. Dunglison. His learning was extensive and varied; he was a ready writer and a voluminous contributor to medical literature. So popular, at one time, were his works, that they were adopted as text-books in nearly all our medical colleges. He was a highly courteous gentleman, and possessed uncommon tact and good sense, which contributed largely to the success of the institution, over the affairs of which he presided. He had many social qualities also which endeared him to a large circle of friends who will long deplore his loss.—*Med. News.*

DIED, in Albany, June 17th, Prof. ALDEN MARCH, aged about seventy-four.

The life of Dr. March is identified with the history of the profession, of which he was one of the brightest ornaments. He was alike distinguished as a scholar, teacher, and as a bold yet cautious and skillful surgeon. Beloved in private life, crowned with the highest honors of the profession, his decease will be widely and deeply lamented. To the Albany Medical College, of which he was the founder, his loss can with difficulty be replaced.

DIED.—In Miramac, Indiana, May 28, 1869, Dr. ASA M. PEARSON, aged thirty-seven years.

Dr. Pearson was a member of the class in Rush Medical College in 1856-7.

